# Uptake of Aortic ^18^F-FDG Is Correlated with Low-Density Lipoprotein Cholesterol and Leptin in a General Population

**DOI:** 10.1371/journal.pone.0111990

**Published:** 2014-11-06

**Authors:** Ai Haraguchi, Naomi Hayashida, Toshihiko Kamasaki, Izumi Miyamoto, Toshiya Usui, Takao Ando, Norio Abiru, Hironori Yamasaki, Kenya Chiba, Takashi Kudo, Atsushi Kawakami, Noboru Takamura

**Affiliations:** 1 Department of Global Health, Medicine and Welfare, Nagasaki University Graduate School of Biomedical Sciences, Nagasaki, Japan; 2 Department of Endocrinology and Metabolism, Nagasaki University Graduate School of Biomedical Sciences, Nagasaki, Japan; 3 Radioisotope Medicine, Nagasaki University Graduate School of Biomedical Sciences, Nagasaki, Japan; 4 Center for Health and Community Medicine, Nagasaki University, Nagasaki, Japan; 5 Nishi-Isahaya Hospital PET/CT Diagnostic Imaging Center, Isahaya, Japan; University Hospital Medical Centre, Germany

## Abstract

**Objective:**

This study investigated the relationship between aortic ^18^F-fluoro-2-deoxy-D-glucose (^18^F-FDG) uptake and clinical and laboratory findings related to atherosclerosis in a general population.

**Methods:**

^18^F-FDG uptake in the ascending aorta was measured on the positron emission tomography/computed tomography (PET/CT) scans of 211 Japanese adults. The maximum target-to-background ratio (TBR) was compared with clinical and laboratory atherosclerosis findings.

**Results:**

By multivariate regression analysis adjusted for age and sex, TBR-ascending aorta (TBR-A) was significantly correlated with various clinical and laboratory parameters, such as body mass index, log visceral fat area, low-density lipoprotein cholesterol (LDL-C), log fasting immunoreactive insulin, log homeostasis model assessment of insulin resistance, log total adiponectin and log-leptin, in all subjects. Furthermore, by multivariate linear regression analysis adjusted for confounding factors, TBR-A was significantly correlated with LDL-C (*β* = 0.001, p = 0.03) and log-leptin (*β* = 0.336, p<0.01) in all subjects.

**Conclusion:**

TBR-A was significantly correlated with LDL-C and log-leptin independent from confounding factors. Our results suggest that aortic ^18^F-FDG uptake is a good marker of atherosclerosis, even in a general population.

## Introduction

Atherosclerotic diseases such as coronary heart disease and cerebrovascular disease are the leading cause of illness and death worldwide [1]. Although controlling risk factors such as smoking, blood pressure, and serum lipid levels [2] to reduce atherothrombotic events is recommended, a fifth of all cardiovascular events occur in individuals with no identifiable traditional risk factors [3]. Recently, atherosclerosis has been recognized as an inflammatory disease [4]. It is well known that plaque composition and biologic activity contribute significantly to the risk of vascular events [5], [6]; however, since angiography is invasive for the evaluation of atherosclerosis, plaque inflammation and vulnerability cannot be directly observed [7].

Collectively, adipose tissue can be perceived a large gland that produces adipocytokines. Some studies have suggested that inflammation and dysfunction of adipose tissue in obesity may induce abnormal production of adipocytokines and result in the higher incidence of cardiovascular diseases among obese people [8]. Adipocytokines such as leptin and adiponectin play important roles in the metabolic regulation of obesity and obesity-related complications.

Leptin is mainly produced by white adipose tissue [9] and its production is regulated by energy level, food intake, several hormones, and various inflammatory mediators [10]. Leptin also provides the functional link between the immune system and energy homeostasis [11]. Hyperleptinemia in the general population is associated with atherosclerosis, hypertension, and metabolic syndrome [10]. Increased serum concentrations of leptin are also associated with an increased risk of myocardial infarction and stroke, independent of obesity and other traditional cardiovascular risk factors [12].

^18^F-fluoro-2-deoxy-D-glucose positron emission tomography (^18^F-FDG-PET) is an imaging modality to detect glucose metabolism in normal and disease states such as malignancies [13]. Recent technological advances have enabled the non-invasive use of FDG-PET imaging within a short period [14], [15]. Although FDG-PET has been mainly used for cancer diagnosis and early detection, it can also detect inflammatory changes in the arterial wall, which may represent early stages of atherosclerosis [16], [17]. Previous studies showed FDG accumulation in atherosclerotic plaques [18]–[20]. However, availability of ^18^F-FDG uptake for the evaluation of the development of atherosclerosis remains unclear.

In this study, we thus evaluated a general population to investigate the relationship between aortic ^18^F-FDG uptake and clinical parameters in the development of atherosclerotic disease.

## Methods

### 1.1. Study subjects

The study was conducted during a cancer-screening program at the Nishi-Isahaya Hospital PET/CT Diagnostic Imaging Center (Nagasaki, Japan). We initially included 253 consecutive study candidates who underwent PET/CT screening for malignancies at the Center between June 2012 and April 2013. Among these subjects, we excluded 42 subjects for following reasons: 2 had glycosylated hemoglobin A_1c_ (HbA_1c_) at >7.0%; 3 had dyslipidemia and low-density lipoprotein cholesterol (LDL-C)>6.48 mmol/L and/or high-density lipoprotein cholesterol (HDL-C)<0.78 mmol/L and/or triglyceride (TG) >3.39 mmol/L; 9 had cerebral infarction or hemorrhage[21]; 13 had apparent chronic kidney disease; 13 had collagen disease, including rheumatoid arthritis; and 2 had current malignant tumors. Ultimately, 211 Japanese participants (119 men and 92 women) were included in the study. None of the participants showed malignancies on PET/CT. The study was approved by the ethics committee of Nagasaki University (project registration number 12053002), and written informed consent was obtained from all participants.

### 1.2. Data collection and laboratory measurements

A questionnaire was administered to evaluate medical history, smoking habits, and drug use. Height and weight were measured, and body mass index (BMI; kg/m^2^) was calculated. Blood pressure (BP) was measured in a supine position using a standard sphygmomanometer.

Blood samples were collected from each participant after overnight fasting for the measurements of lipid profile, fasting plasma glucose (FPG), fasting immunoreactive insulin (IRI), HbA_1c_ as defined by the National Glycohemoglobin Standardization Program (NGSP), 1,5-anhydroglucitol (1,5-AG), creatinine (Cr), uric acid (UA), high sensitive C-reactive protein (hs-CRP), high-molecular-weight adiponectin (HMW-A), total adiponectin (T-A), and leptin. Insulin resistance was estimated using the homeostasis model assessment of insulin resistance (HOMA-IR) from FPG and IRI concentrations using the following formula: HOMA-IR  =  (fasting IRI [µU/ml] × FPG [mg/dl])/405. Estimated glomerular filtration rate (eGFR) was calculated using creatinine, age, and sex [22].

Serum T-A, HMW-A, leptin concentrations were measured using commercial kits, and visceral fat area was measured by body fat meter.

### 1.3. ^18^F-FDG-PET/CT imaging

The PET/CT imaging study was performed with a PET/CT scanner (Discovery ST; GE Healthcare, Milwaukee, Wisconsin, USA). Details of this procedure have been described elsewhere [23]–[25]. In brief, imaging was started 50 min after intravenous injection of ^18^F-FDG through an anterior cubital vein. Injected ^18^F-FDG doses were determined according to each subject's weight (4 MBq/kg); individual doses ranged from 146.2–403.8 MBq. The patients fasted for at least 6 h before ^18^F-FDG injection, and the glucose level of each participant was measured using a simple method before ^18^F-FDG injection. All participants showed glucose levels lower than 11.10 mmol/L (3.66–8.94). Each participant was scanned in the supine position with his or her arms placed alongside the body. The image acquisition time was 2.5 min per bed position. There were 7–8 bed positions, which allowed scanning of the entire body in a three-dimensional mode. The acquisition parameters for dual-detector helical CT were 140 kV, 30 mA, 3.75-mm slice thickness, and a pitch of 1.5. Attenuation correction was performed with acquired CT data. The PET/CT images displayed as coronal, sagittal, and transaxial slices were viewed on dedicated Workstation (Xeleris, GE Healthcare) [23].

### 1.4. Image Analysis

**Figure 1:**
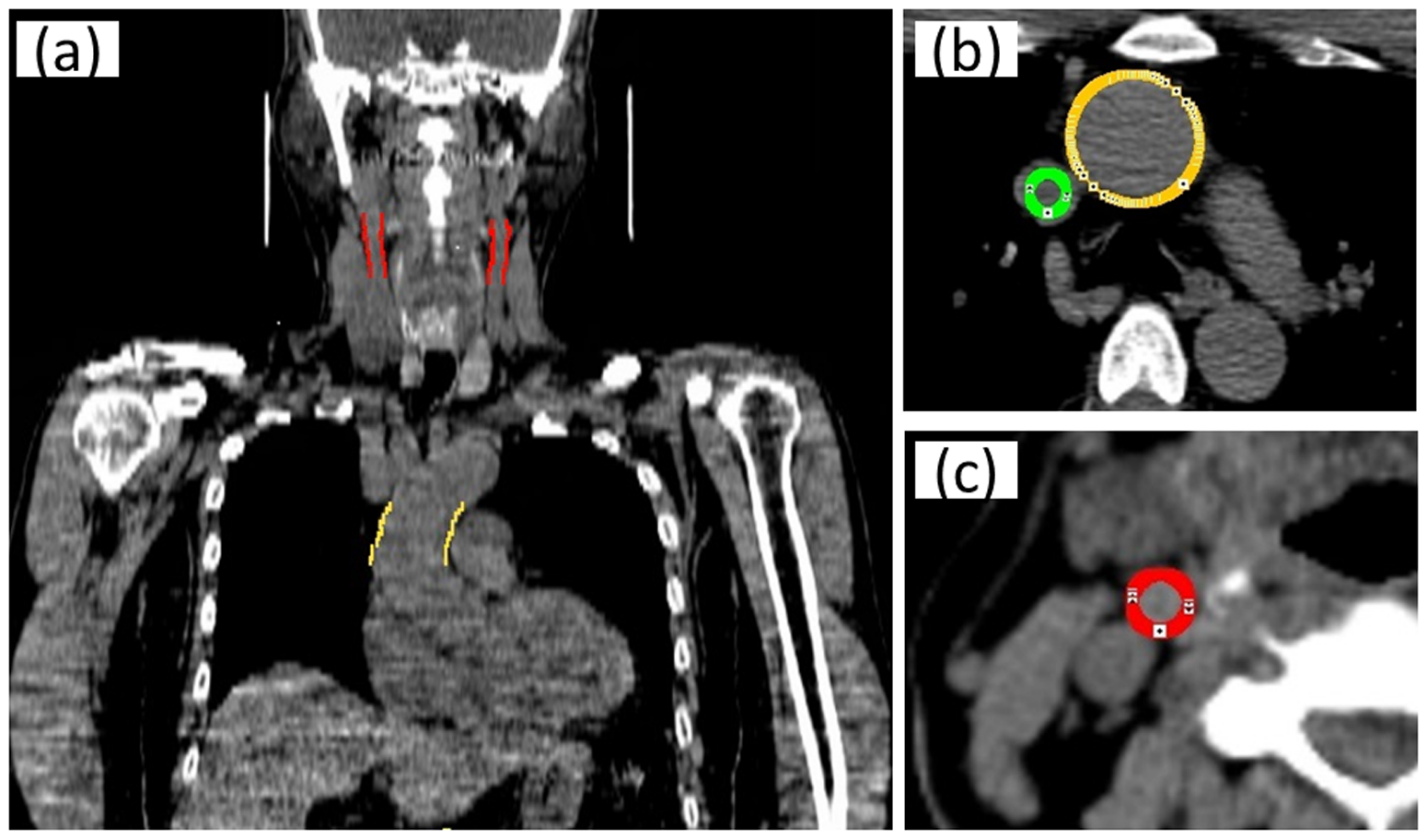
Diagrammatic representation of how to place the ROIs on PET/CT image of a 63-year-old man. (a) Coronal section with ROI. (b) Axial section with ROI of superior vena cava and ascending aorta. (c) Axial section with ROI of right carotid artery.

The PET and CT images of each participant were analyzed using a dedicated image analyzing software on a personal computer. The PET and CT images were superimposed to form a fused PET/CT image using the program. For the index of glucose metabolic activity in the common carotid arteries and ascending aorta, we calculated standardized uptake values (SUVs) of ^18^F-FDG in each region of interest (ROI). The ROIs of the common carotid arteries were obtained from 10 consecutive tomographic slices, beginning from the merging point with the carotid bifurcation [20]. The ROIs of the ascending aorta were also obtained from 10 consecutive tomographic slices, beginning from the merging point with the aortic arch. Maximum SUVs were obtained from each ROI. Blood-pool activity was measured by placing a circular ROI in the mid lumen of the superior vena cava on two different tomographic slices and then averaging the values to obtain the background SUV. The maximum SUVs of each arterial segment were averaged for both carotid arteries and the ascending aorta, and were then divided by the background SUV to yield maximum target-to-background ratios (TBR) for each patient [26] (Figure 1).

Prior to the analysis, we evaluated the correlation between TBR-ascending aorta (TBR-A) and other TBRs, such as TBR-right common carotid artery, TBR-left common carotid artery, TBR-right carotid bifurcation, and TBR-left carotid bifurcation, in 60 randomly selected subjects. We confirmed high correlations (r = 0.64, p<0.01 vs. TBR-right common carotid artery, r = 0.65, p<0.01 vs. TBR-left common carotid artery, r = 0.60, p<0.01 vs. TBR-right carotid bifurcation and r = 0.59, p<0.01 vs. TBR-left carotid bifurcation). Based on these results, TBR-A was used for analysis in this study.

### 1.5. Statistical Analysis

Results are expressed as mean ± standard deviation (SD). Differences in continuous values between men and women were compared using the Mann-Whitney U test, and differences between categorized values were evaluated using the *χ* ^2^-test. As visceral fat area, TG, IRI, HOMA-IR, 1,5-AG, HMW-A, T-A, leptin, and hs-CRP showed a skewed distribution, logarithmic transformation was performed for multivariate linear regression analysis. Simple correlations of TBR-A with other variables were evaluated using univariate linear regression analysis. The correlations of TBR-A adjusted for age, sex, and other confounding factors (log visceral fat area, LDL-C, log HOMA-IR, and log leptin) were evaluated by multivariate linear regression analysis. A *P*-value <0.05 was considered statistically significant. All statistical analyses were performed using statistical software.

## Results

The characteristics of the study subjects are shown in **Table 1**. Subjects had a mean age (±SD) of 54.4±8.9 years (range, 34–75 years) in 119 men. For the 92 women, the mean age was 56.5±10.9 years (range, 33–87 years) (p = 0.38). TBR-A of men (1.74±0.39) was significantly higher than that of women (1.54±0.20) (p<0.01).

**Table 1:** Characteristics of the Study Population.

	Men (n = 119)	Women (n = 92)	All (n = 211)	*p*
Age (years)	54.4±8.9	56.5±10.9	55.3±9.9	0.38
BMI^a^ (kg/m^2^)	24.1±3.0	22.2±3.0	23.3±3.2	<0.01**
Waist circumference (cm)	85.5±9.7	82.0±8.9	84.0±9.5	0.02*
Visceral fat area (cm^2^)	116.4±36.4	79.6±29.4	100.4±38.1	<0.01**
Systolic BP^b^ (mmHg)	124.2±12.7	117.3±12.6	121.2±13.1	<0.01**
Diastolic BP^b^ (mmHg)	79.6±9.0	72.0±10.0	76.3±10.2	<0.01**
FPG^c^ (mmol/L)	5.48±0.71	5.04±0.40	5.28±0.63	<0.01**
HbA_1c_^d^ (NGSP) (%)	5.5±0.4	5.4±0.3	5.5±0.4	0.58
LDL-C (mmol/L)	3.05±0.78	3.45±0.93	3.22±0.87	<0.01**
HDL-C (mmol/L)	1.47±0.34	1.68±0.34	1.56±0.36	<0.01**
TG (mmol/L)	1.29±0.74	0.97±0.41	1.15±0.64	<0.01**
UA^e^ (µmol/L)	362.86±71.38	261.73±59.49	315.27±83.28	<0.01**
Cr^f^ (µmol/L)	70.72±10.61	53.04±8.84	62.76±13.26	<0.01**
eGFR^g^ (mL/min/1.73 m^2^)	81.3±14.0	81.5±14.5	81.4±14.2	0.63
TBR-Aorta^h^	1.74±0.39	1.54±0.20	1.65±0.33	<0.01**
IRI^i^ (pmol/L)	32.64±26.25	25.90±14.45	29.72±22.09	0.20
HOMA-IR^j^	1.18±0.99	0.85±0.51	1.03±0.83	0.04*
1,5-AG^k^ (µmol/L)	138.28±61.53	115.14±46.91	127.93±56.65	<0.01**
HMW-A^l^ (mg/L)	3.47±2.12	5.91±3.18	4.53±2.89	<0.01**
T-A^m^ (mg/L)	8.4±3.5	13.3±5.9	10.6±5.3	<0.01**
Leptin (µg/L)	2.97±1.83	5.68±3.05	4.15±2.78	<0.01**
hs-CRP^n^ (nmol/L)	8.92±16.42	11.29±48.56	9.95±34.27	<0.01**
Anti-hypertensive agents	20.2%	19.8%	20.0%	0.94
Anti-diabetic agents	4.2%	2.2%	3.3%	0.42
Statin	10.1%	6.6%	8.6%	0.37
Lipid-lowering agents	14.3%	7.7%	11.4%	0.14
Antiplatelet agents	8.4%	3.3%	6.2%	0.13
Gout remedy	8.4%	0%	4.8%	<0.01**
Smoking history				<0.01**
none	24.6%	85.9%	51.4%	
current smoke	30.5%	5.4%	19.5%	
past smoke	44.9%	8.7%	29.0%	

**; p<0.01, *; p<0.05.

a body mass index, ^b^ blood pressure, ^c^ fasting plasma glucose, ^d^ glycosylated hemoglobin A_1c_, ^e^ uric acid, ^f^ creatinine, ^g^ estimated glomerular filtration rate, ^h^ target-to-background ratio-ascending aorta, ^i^immunoreactive insulin, ^j^ homeostasis model assessment of insulin resistance, ^k^ 1,5-anhydroglucitol, ^l^ high-molecular-weight adiponectin, ^m^ total adiponectin, ^n^ high sensitive C-reactive protein.

**Table 2** shows the correlation coefficients of TBR-A with other variables as determined by univariate regression analysis. TBR-A was significantly correlated with BMI, waist circumference, visceral fat area, diastolic BP, FPG, TG, UA, Cr, IRI, HOMA-IR, HMW-A, T-A, and hs-CRP in all subjects. TBR-A was significantly correlated with BMI, IRI, HOMA-IR, and leptin in both men and women. Furthermore, TBR-A was significantly correlated with UA in men and waist circumference in women.

**Table 2:** Correlation Coefficients of the TBR-Aorta with Other Variables as Determined by Univariate Regression Analysis.

	Men (n = 119)	Women (n = 92)	All (n = 211)
Age	−0.047	0.065	−0.019
BMI	0.264**	0.244*	0.318**
Waist Circumference	0.179	0.207*	0.224**
Visceral Fat Area	0.125	0.204	0.235**
Systolic BP	−0.008	−0.019	0.065
Diastolic BP	0.098	0.056	0.196**
FPG	0.011	0.094	0.144*
HbA1c	0.070	−0.012	0.041
LDL-C	0.168	0.151	0.090
HDL-C	0.019	−0.007	−0.079
TG	0.145	0.044	0.165*
UA	0.218*	0.063	0.300**
Cr	0.111	0.189	0.276**
eGFR	−0.079	−0.181	−0.120
IRI	0.202*	0.209*	0.205**
HOMA-IR	0.183*	0.214*	0.206**
1,5-AG	−0.092	0.136	0.057
HMW-A	−0.129	−0.179	−0.265**
T-A	−0.126	−0.181	−0.264**
Leptin	0.244**	0.369**	0.078
hs-CRP	0.149	0.106	0.176*

**; p<0.01, *; p<0.05.

By multivariate regression analysis adjusted for age and sex, TBR-A was significantly correlated with BMI, waist circumference, log visceral fat area, diastolic BP, LDL-C, log TG, UA, Cr, log IRI, log HOMA-IR, log T-A, log-leptin, and log hs-CRP in all subjects (**Table 3**).

**Table 3:** Multiple Linear Regression Analysis of TBR-Aorta Adjusted for Age and Sex in All Subjects.

	β	95%CI	*p*
BMI	0.036	0.022, 0.049	<0.01**
Waist Circumference	0.008	0.003, 0.013	<0.01**
log Visceral Fat Area	0.440	0.140, 0.740	<0.01**
Systolic BP	0.001	−0.003, 0.005	0.52
Diastolic BP	0.005	0.000, 0.009	<0.05*
FPG	0.002	−0.002, 0.006	0.34
HbA1c	0.052	−0.065, 0.169	0.38
LDL-C	0.002	0.001, 0.003	<0.01**
HDL-C	0.000	−0.004, 0.003	0.78
log TG	0.300	0.088, 0.513	<0.01**
UA	0.047	0.009, 0.085	0.02*
Cr	0.431	0.030, 0.832	0.04*
eGFR	−0.003	−0.007, 0.000	0.06
log IRI	0.099	0.003, 0.195	0.04*
log HOMA-IR	0.100	0.006, 0.194	0.04*
log 1,5-AG	0.010	−0.076, 0.095	0.82
log HMW-A	−0.130	−0.302, 0.042	0.14
log T-A	−0.242	−0.482, −0.002	<0.05*
log leptin	0.357	0.196, 0.518	<0.01**
log hs-CRP	0.107	0.021, 0.194	0.02*

**; p<0.01, *; p<0.05.

β: standardized regression coefficient.

95%CI: 95% confidence interval.

Furthermore, by multivariate linear regression analysis adjusted for confounding factors, TBR-A was significantly correlated with LDL-C (*β* = 0.001, p = 0.03) and log leptin (*β* = 0.336, p<0.01), as were age and sex, in all subjects (**T**able **4**).

**Table 4:** Multiple Linear Regression Analysis Adjusted by Confounding Factors.

	β	95%CI	*p*
Age	−0.005	−0.010, 0.000	0.03*
Sex	−0.260	−0.390, −0.131	<0.01**
log Visceral Fat Area	0.247	−0.058, 0.553	0.11
LDL-C	0.001	0.000, 0.003	0.03*
log HOMA-IR	−0.042	−0.163, 0.080	0.50
log leptin	0.336	0.112, 0.560	<0.01**

**; p<0.01, *; p<0.05.

## Discussion

By multivariate regression analysis adjusted for age and sex, we showed that TBR-A was significantly correlated with various clinical and laboratory parameters, such as BMI, log visceral fat area, LDL-C, log TG, log IRI, log HOMA-IR, log T-A and log-leptin. Furthermore, by multivariate linear regression analysis adjusted for confounding factors, we showed that LDL-C and log-leptin were significantly correlated with TBR-A.

Recent studies reported that ^18^F-FDG PET could be a useful tool for the measurement of vascular inflammation. Histological studies by Rudd et al. indicated that FDG accumulation was localized within the atherosclerotic plaques [18]. Tawakol et al. showed that FDG-PET imaging can determine the *in vivo* macrophage content of carotid plaques [19], while Tahara et al. showed strong FDG accumulation in the carotid artery in an atherosclerotic plaque lesion [20]. Further studies confirmed that FDG uptake by the carotid artery wall was associated with atherosclerotic markers in healthy individuals [26]–[28]. In this study, we showed that TBR-A was significantly associated with BMI, which is consistent with previous studies [20], [27].

Univariate regression analysis revealed a significant relationship between TBR-A and leptin in both men and women, but not in all subjects. A greater serum leptin level has been reported in women than in men [29], which is consistent with our current study findings. These different profiles of leptin levels for men and women resulted in the non-significant correlation observed by univariate regression analysis between TBR-A and leptin in all subjects in the present study.

On the other hand, we first reported that TBR-A was significantly associated with leptin after adjusting for confounding factors. Hyperleptinemia in the general population is associated with atherosclerotic disease and their risk factors [10], [12]. This may be partly explained by the fact that increased leptin leads to increased insulin resistance, homeostasis imbalance, and vascular inflammation [30]. Dubey et al. reported that leptin and hs-CRP were independent predictors for complex lesion morphology in unstable angina [31]. Leptin is reported to play an important role in the early stages of atherosclerosis development by initiating leukocyte and macrophage recruitment to the endothelial wall [10]. Schneiderman et al. also showed that neurologically symptomatic patients overexpress leptin mRNA and synthesize leptin protein in carotid plaque macrophages and smooth muscle cells. This induction might be activated by cytokines like TNF-α when these are available within the plaque and in the systemic circulation. Excessive synthesis of leptin within the plaque could have paracrine or autocrine effects that could lead to destabilization of the atherosclerotic lesion, resulting in embolic complications [32]. In combination with our results, these suggest that leptin is a biomarker of active plaque. We also found that TBR-A is significantly correlated with LDL-C after adjusting for confounding factors. It is well known that high serum levels of LDL-C play a main role in the initiation and progression of atherosclerosis [33]. The nascent plaque progresses over time to form a raised lesion consisting of a fibrous layer of scar tissue overlying a lipid-rich core. These raised fibrous plaques then progress at a rate that is proportional to the circulating level of plasma LDL-C to ultimately form larger and more complex lesions, which continue to progress and can eventually become vulnerable to disruption [34], [35]. Tahara et al. showed that LDL-C-lowering therapy with statin attenuates plaque inflammation evaluated by FDG-PET [36]. Further studies are needed to clarify the relationship between aortic ^18^F-FDG uptake and lipid metabolism.

Montecchi et al. reported that type 2 diabetes caused atherosclerosis at a more accelerated rate than hypercholesterolemia [37]. However, we could not find a significant relationship between TBR-A and markers related with glycometabolism such as HbA_1c_ and HOMA-IR. This is probably because only 3.3% of the patients in this present study were diabetic, and the subjects whose HbA_1c_ was >7% or whose glucose levels were higher than 11.10 mmol/L were excluded. It may be difficult to assess the influence of hyperglycemia to vascular inflammation by FDG-PET, since the FDG uptake rate in tissues is enhanced by high plasma glucose levels [38], [39].

One interesting finding was that multivariate linear regression analysis of 152 of 211 subjects with no treatment for atherosclerotic risk factors revealed a significant correlation between TBR-A and log leptin (*β* = 0.623, p<0.01), but not with LDL-C (*β* = 0, p = 0.80). This may suggest that a general population with no treatment for atherosclerotic risk factors is less likely to have progressive atherosclerotic disease.

There are some limitations to this study. There may have been a selection bias, as individuals who participated in cancer screening by PET/CT were enrolled in the study. The sample size was relatively small, and other non-invasive methods for atherosclerosis, such as carotid intima-media thickness or ankle-brachial index, could not be evaluated. We were also unable to assess calcium score because we obtained these images without cardiac gating and without the patients holding their breath. Further studies are needed to clarify the availability of aortic ^18^F-FDG uptake to clinical and laboratory findings related with atherosclerosis.

In conclusion, TBR-A was significantly correlated with LDL-C and log-leptin, independent from confounding factors. Our results suggest that aortic ^18^F-FDG uptake is a good marker of atherosclerosis, even in a general population.
